# Sustained release of linezolid in ocular insert based on lipophilic modified structure of sodium alginate

**DOI:** 10.22038/ijbms.2021.49866.11385

**Published:** 2021-03

**Authors:** Ashkan MohammadSadeghi, Fatemeh Farjadian, Shohreh Alipour

**Affiliations:** 1 Department of Quality Control, School of Pharmacy, Shiraz University of Medical Sciences, Shiraz, Iran; 2 Pharmaceutical Sciences Research Center, Shiraz University of Medical Sciences, Shiraz, Iran

**Keywords:** Copolymer, Grafting, Linezolid, Ocular insert, Sodium alginate

## Abstract

**Objective(s):**

Ocular inserts are usually polymeric thin films with increased ocular residence time and sustained drug release capacity. Sodium alginate is a biocompatible and biodegradable carrier; however, initial burst release of encapsulated drug within it, is recognized as a challenge. Grafting –addition of functional moieties to a polymer– is a technique to modify polymers’ physicochemical properties, including higher ability to control drug release. Linezolid (LNZ) solution is used in consecutive doses in treatment of antibiotic-resistant Gram-positive bacterial infections especially induced by methicillin resistant *Staphylococcus aureus* (MRSA).

**Materials and Methods:**

Grafted alginate copolymers were synthesized using butyl methacrylate (BMC) and lauryl methacrylate (LMC) at two different reaction times (12 hr and 24 hr). Copolymerization was evaluated by ^1^H-NMR, Ft-IR, and TGA. Copolymer safety was examined by cytotoxicity test against HEK-293 cell. Linezolid inserts were prepared using optimized copolymers and characterized.

**Results:**

^1^H-NMR, Ft-IR, and TGA confirmed the successful grafting of alginate copolymers. ALG-B24 and ALG-L12 showed the highest safety against HEK-293 cell line comparing with intact alginate. Linezolid insert characterization results indicated a slower linezolid release profile related to creation of a lipophilic structure. A better strength property for linezolid loaded ALG-B24 and ALG-L12 inserts was obtained while ALG-L12 showed a stronger adhesive force compared with intact alginate. Antibacterial efficacy on clinical isolated MRSA after 24 hr was similar to linezolid solution.

**Conclusion:**

Lipophilic alginate copolymer (ALG-L12) showed a sustained release capability while retaining its main feature in strong film forming ability so it seems to be a promising safe carrier.

## Introduction

Eyes are wonderful sensory organs through which most of the environmental information is received by observing. Ocular drug delivery considering physiological restrictions inflicted by protective mechanism of the eye can lead to a low absorption amount, and sometimes short duration of therapeutic effects. Ocular drug delivery is one of the most interesting and challenging topics over the past years. The most important limitation of conventional ocular drug delivery systems is the low precorneal contact time and consequently low absorption. New ocular drug delivery systems offer higher efficacy with the lowest harm (1). The physiology, anatomy, and biochemistry of the eye have created a non-penetrating defensive barrier against outside parameters. Overcoming these eye-protecting barriers without affecting permeable tissues is one of the challenges facing drug delivery systems (2).

New non-invasive ocular delivery system allows the drug to target the frontal chamber, while it may lower drug systemic adverse effects, as well (3). Ocular inserts are thin polymeric films that can be placed in the conjunctival sac (4). Ocular inserts benefits over conventional dosage forms include increment of ocular residence time, facility of releasing drugs at a steady rate, precise dosing, increased shelf life, and reduced systemic absorption (2, 5). 

Gram-positive pathogens, and especially methicillin resistant *Staphylococcus aureus* (MRSA) are mostly the reason for common non-contact lens related endophthalmitis (6, 7). Bacterial endophthalmitis treatment is a medical emergency status, since a delayed treatment may cause irreversible defects in the patients’ vision (8). Drug penetration through blood-retinal barrier is the most challenging obstacle for increasing drug concentration in vitreous, especially in inflammatory states (8, 9). Considering the importance of achieving inhibitory concentration in vitreous fluids and aqueous medium and also for endophthalmitis, systemic treatments seem to be inefficient (8). There are a few antibiotics (imipenem, ofloxacin, levofloxacin, gatifloxacin, and some cephalosporins) that have been reported to penetrate into the vitreous cavity. Generally, aminoglycoside and vancomycin showed poor intravitreal penetration, which can lead to invasive treatment including direct intravitreal injection of antibiotics (8, 9). 

Along with the reported clinical cephalosporin and fluoroquinolone-resistant organism, vancomycin leaves can be used as the gold standard in combination therapy for severe and resistant Gram-positive MRSA infections (9, 10). Topical vancomycin showed another problem during the treatment, even at reduced concentrations (liquid 0.2%) while it was mostly painful and may be toxic in frequent administration to the ocular surface (6). Therefore, due to vancomycin’s poor intravitreal penetration and side effects, even in topical uses and considering the reported vancomycin resistant organisms, it is necessary to introduce some alternative antibiotics (6, 7, 10, 11). 

Linezolid (LNZ) is an oxazolidinone with narrow activity specified against Gram-positive bacteria. LNZ is also active against the most clinically significant Gram-positive pathogens including MRSA with lower minimum inhibitory concentrations (MICs) compared with the currently used antibiotics. LNZ is an antibiotic with a unique mechanism of action in inhibition of protein synthesis, which can lower the risk of cross resistance along with other protein-synthesis inhibitors (8, 11). Previous studies reported better patient toleration of topical LNZ compared with vancomycin with lower toxicity (7) and significant LNZ intravitreal penetration after systemic administration (8); therefore, it seems that LNZ can be a good drug to be selected.

Previous studies showed that LNZ was an effective replacement antibiotic for treating Gram-positive bacteria induced keratitis. Another positive note was about the topical LNZ effectiveness on treating a resistant form of corneal infection while vancomycin was ineffective or has not been tolerated (10). Considering exclusive LNZ mechanism of action against bacteria in protein synthesis inhibition, the chance of cross-resistance is very low (6, 10, 12). 

Besides, animal pharmacokinetic studies showed that LNZ has good corneal, vitreous, and retinal tissue penetration with the lowest toxicity (7). Also, considerable linezolid concentration has been reported in human vitreous and aqueous humor after intravenous or oral administration (12). Human studies also reported comparable linezolid effectiveness and tolerance on treating MRSA-induced eye infections with vancomycin (6). The hourly administered LNZ eye drop’s effectiveness on Gram-positive microbes induced keratitis was demonstrated in animals (12) and humans (10, 12); however, there is not enough development regarding LNZ ocular formulations.

One of the most common causes of ocular infections is considered to be Gram-positive bacteria. LNZ is an antibiotic of the oxazolidinone group, which is used in the treatment of infection by antibiotic-resistant Gram-positive bacteria (13). Previous studies reported successful LNZ treatments for corneal infections compared with vancomycin (7). The most common pathogen reported in all ocular infections is MRSA. Previous studies have also confirmed higher tolerance and effectiveness of local LNZ on reducing streptococcal colonies to treat corneal infections in rabbits compared with vancomycin (11). 

Sodium alginate is a non-toxic, biodegradable, and biocompatible polymer that is used as a carrier in drug delivery systems especially in ocular formulations (14, 15); however, initial burst release or rapid release of encapsulated drug in alginate polymer is one of the most difficult challenges in sustained drug delivery systems (15-19). In order to reduce the burst release, different approaches have been examined, which may include coating with polycations (16) or increasing the polymer hydrophobicity by grafting polymers (19). 

Also, Grafting is one of the well-established and successful methods of polymer modification (20, 21). Moreover, it is a technique for addition of functional moieties to a polymer, which may provide a widespread species of polymers with the modified physicochemical properties (17, 19). Indeed, polymer grafting is an interesting approach for transferring functional groups into a polymeric backbone as side chains. Polymer grafted alginates (graft co-polymerization) have a higher ability in controlling drug release compared with intact polymers (19). One of the most common types of polymerization reactions is the free radical method, which is considered as the most applicable one for vinyl monomers. Polymers that are constructed in this way contain polystyrene, poly (methyl methacrylate), acrylamide, poly (vinyl acetate), and branched polyethylene (22). Grafting co-polymerization has been extensively applied in the preparation of the modified structures of alginate with numerous applications in drug delivery. Previous reports indicated a prolonged release for encapsulated drug in butyl and methyl methacrylate alginate grafted microspheres (19). 

The novelty of the present study was preparing alginate grafted poly(butyl methacrylate) and poly(lauryl methacrylate), and also evaluating their safety on human kidney epithelial cells, to obtain a suitable polymer for thin film preparation, which should be characterized as ocular insert with a slower release pattern of LNZ. 

## Materials and Methods

***Materials***

LNZ was purchased from Exir Pharmaceutical Company, Iran. Sodium alginate was purchased from Samchun, Korea. Butyl methacrylate (BMC), lauryl methacrylate (LMC), and potassium persulfate were purchased from Merck, Germany. Dulbecco’s modified Eagle medium (DMEM), fetal bovine serum (FBS), and penicillin-streptomycin were provided from Biosera, Ringmer, UK. MTT, i.e., 3-(4,5-Dimethylthiazol-2-yl)-2,5-diphenyltetrazolium bromide, was purchased from Sigma, USA. The solvents were purchased locally in analytical grade. HEK-293 cell line was purchased from Iranian biological resource center (IBRC).

***Synthesis of sodium alginate grafted copolymers***

Sodium alginate-grafted- poly (butyl methacrylate) and sodium alginate-grafted-poly (lauryl methacrylate) were synthesized. Briefly, 22 mmol potassium persulfate was added to ALG 4% ethanolic solution and stirred at room temperature, then BMC (0.02 mM) or LMC (0.2 mM) were added and stirred at 70 °C. Different copolymers (ALG-g-BMC and ALG-g-LMC) were synthesized at two different reaction times of 12 hr and 24 hr. Copolymers were purified with 20 ml diethyl ether and ethanol three times and the remaining product centrifuged to remove contaminants and unnecessary by-products of the reaction, the final copolymer powders were obtained by freeze drying. 

***Characterization of sodium alginate grafted copolymers***

***In vitro toxicity test***

To evaluate all synthesized copolymers’ safety, cell cytotoxicity was evaluated using MTT assay, which was performed on human kidney epithelial cells, i.e., the HEK-293 cell line. Cells were cultivated in growth media of DMEM containing 10% FBS and 1% penicillin/streptomycin, which was maintained at 37 °C in a humidified environment and 5% CO_2_. In a 96-well plate, 10000 cells per well were seeded and after 24 hr incubation, each cell was treated with ALG, ALG-B12, ALG-B24, ALG-L12, and ALG-L24 at concentration of 2%. After 24 hr, 30 μl of MTT in phosphate buffer solution (5 mg/ml) was added and cells were incubated for 4 hr at 37 °C in a humidified environment and 5% CO_2_. Then, the MTT solution was removed from the wells and 10 μl DMSO was added to plates and shaken for 5 min. Absorptions were determined by a microplate reader at 570 nm. Untreated control cells were assumed to have 100% viability (23).

***Nuclear magnetic resonance (NMR)***

The NMR spectrum shows copolymer synthesis properties and can confirm the validity of synthesis. Considering cytotoxicity results, the composition of the selected safer grafted copolymers ALG-B24, ALG-L12, ALG+BMC, and ALG+LMC physical mixtures were assessed by ^1^H-NMR spectroscopy (Bruker Avance III/400 MHZ, Germany) using D_2_O as solvent.

***Thermoanalysis***

For better evaluation, thermogravimetric analysis (TGA) and differential thermal analysis (DTA) (Mettler Toledo, Italy) of ALG and selected safer grafted copolymers (ALG-B24 and ALG-L24) in addition to ALG+BMC and ALG+LMC physical mixtures were determined with heating rate of 10 °C per min up to 650 °C. The void aluminum pan was sealed as the reference control (24).

***Ft-IR***

The Fourier transform infrared spectroscopy (Ft-IR) was performed using Shimadzu-8400 S (Japan) FTIR equipment (25, 26). Spectra of ALG and selected safer grafted copolymers (ALG-B24 and ALG-L24) powders in addition to ALG+BMC and ALG+LMC physical mixtures were obtained using the KBr pellet method. KBr disks were scanned over a wave number region of 400 to 4000 cm^-1^. 

***LNZ analysis***

LNZ quantification was performed using UV-Vis spectrophotometry method at maximum absorbance wavelength of 251 nm in phosphate buffer solution at pH 7.4 (PBS, 7.4). 

***Analysis method validation***

LNZ calibration curve was constructed with seven different concentrations (2, 4, 8, 10, 12, 16, and 20 μg/ml) that were prepared in three different days. Each sample was tested in triplicate. Calibration curve was validated by linearity, intraday, and inter-day precision, accuracy, limit of detection (LOD), and limit of quantitation (LOQ) (27). 

***Ocular insert preparation***

Considering cell cytotoxicity results, selected polymers were used to prepare ocular inserts using the solvent casting method (28). Polymers were dissolved in distilled water and stirred until reaching a clear solution to obtain final concentration of 2% w/v, then 10% glycerol was added as plasticizer and mixed. LNZ aqueous solution (0.2 % w/v) was then added and mixed to prepare a uniform solution. Ten milliliters of the polymeric solution was poured on a petri-dish, one milliliter aqueous calcium chloride 2% was added and mixed then allowed to dry at room temperature for 24 hr. The dried discs were wrapped in an aluminum foil and stored. 

***Ocular inserts evaluation***

***Thickness measurement***

Thickness of ocular inserts was measured by a digital caliper and recorded in millimeters. All measurements were performed in triplicate and the mean of measurements was reported.

***pH determination***

To determine inserts’ pH, small pieces of inserts (diameter of 1 cm) were swelled in five milliliters distilled water in a closed Petri dish for one hour at room temperature for complete dissolution. The pH of the obtained solution was measured in triplicate.

***Drug content***

The uniformity of the LNZ in the ocular inserts was determined. Small pieces of inserts (diameter of 1 cm) were placed in five milliliters of PBS, pH 7.4, and stirred till complete dissolution. LNZ was extracted using methanol and the LNZ amount was determined using the validated UV-spectrophotometry analysis method. All experiments were tested in triplicate. The practical LNZ amount was compared with its theoretical amount and reported as drug content%.

***In vitro release***

LNZ release from selected ocular inserts was determined using Franz diffusion cell. Small pieces of inserts (diameter of 2 cm) which contained 1 mg LNZ, were applied as membrane between donor and receptor parts, and 25 milliliters PBS, pH 7.4, was the receptor compartment of diffusion cell while it was stirred at 1000 rpm at 37±0.5 °C to prevent diffusion layer effects. At determined times 5, 10, 15, 30, 60, 120, 180, and 240 min, samples were withdrawn and the amount of LNZ was determined using the validated analysis method. Different release models including zero and first-order models and Higuchi release regression coefficients were used to select the best-fitted release model.

***Mechanical properties***

Tensile strength, adhesiveness, and hardness of inserts were measured using TA-DGA and TA11/1000 probe of a Texture Analyzer (CT-3/10,000 Brookfield, USA). For tensile strength, inserts were fixed between the two clamps for 60 sec and at the trigger load of 0.05 N, the upper clasp was pulled apart at a distance of 6 mm and a speed of 2.0 mm/sec while the lower clasp was held immobile. The force at the point the insert broke was recorded as tensile strength. Hardness and adhesiveness were measured using the Texture Pro CT software package. All measures were repeated three times.

***Antibacterial efficacy***

According to a previous study, the MRSA detection test was done using the disk diffusion test at aqueous LNZ concentration of 30 µg/ml for MRSA detection, then antibacterial effectiveness of ALG-L12-LNZ versus ALG-LNZ inserts (with a diameter of one cm) was assessed using agar diffusion test. Clinical isolated methicillin-resistant *S. aureus *(*MRSA *Gp-0408) was seeded in sterile nutrient agar, then the optimized inserts were placed on it, and plates were incubated at 37±0.5 °C for 24 hr. To test antibacterial efficiency the inhibition zone area of inserts was measured around each sample using a ruler at 4, 12, and 24 hr and compared with LNZ solution as a positive control. Each sample was tested in triplicate. Sterile nutrient agar plate was the negative control through the study (29).

***Statistical analysis***

All experiments were done in triplicate (n=3) and data were expressed as the mean±standard deviations. Analysis of variance (ANOVA) via SPSS v.15 software was used for statistical analysis in which a *P*-value of <0.05 was considered to denote a statistically significant difference. 

## Results

***Synthesis of sodium alginate grafted copolymers***

**Figure 1:**
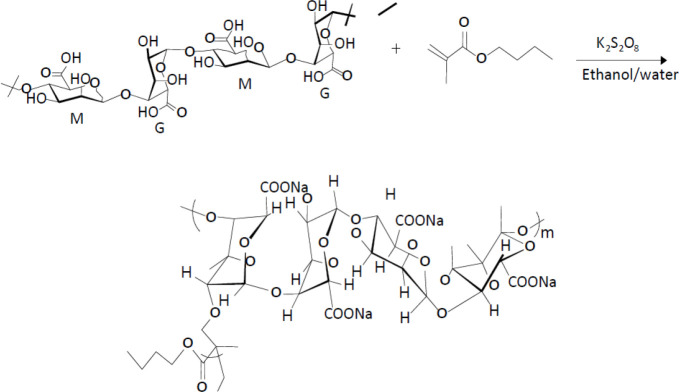
Synthesis of sodium alginate grafted copolymers

The alginate grafted poly(butyl-methacrylate) (ALG-g-BMC) and poly(lauryl-methacrylate) (ALG-g-LMC) were prepared at different reaction times (12 and 24 hr). Figure 1 presents the Synthesis of sodium alginate grafted copolymers scheme.

***Characterization of sodium alginate grafted copolymers***

***Cell cytotoxicity study***

**Figure 2:**
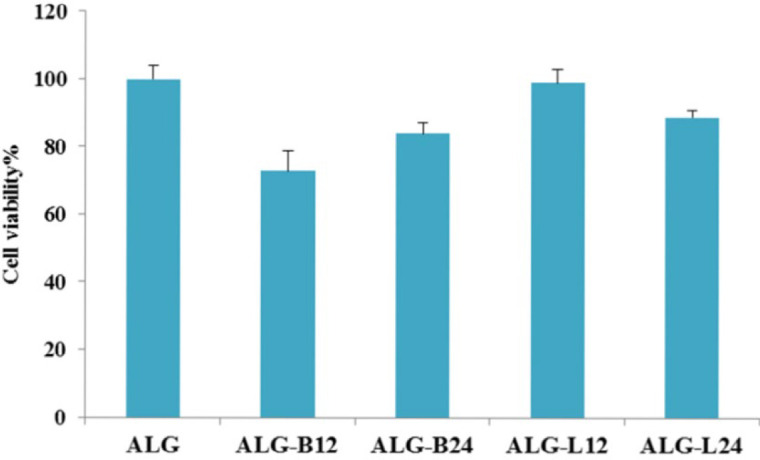
Cell cytotoxicity effect of copolymers (n=3)

Sodium alginate grafted copolymers’ safety was determined using cell cytotoxicity study. As seen in Figure 2, ALG-B24 and ALG-L12 showed the lowest cytotoxicity in their group compared with alginate as a non-toxic polymer (*P*≤0.05). Therefore, they were selected as safer copolymers for further evaluation.

^1^ ***H-NMR***

**Figure 3:**
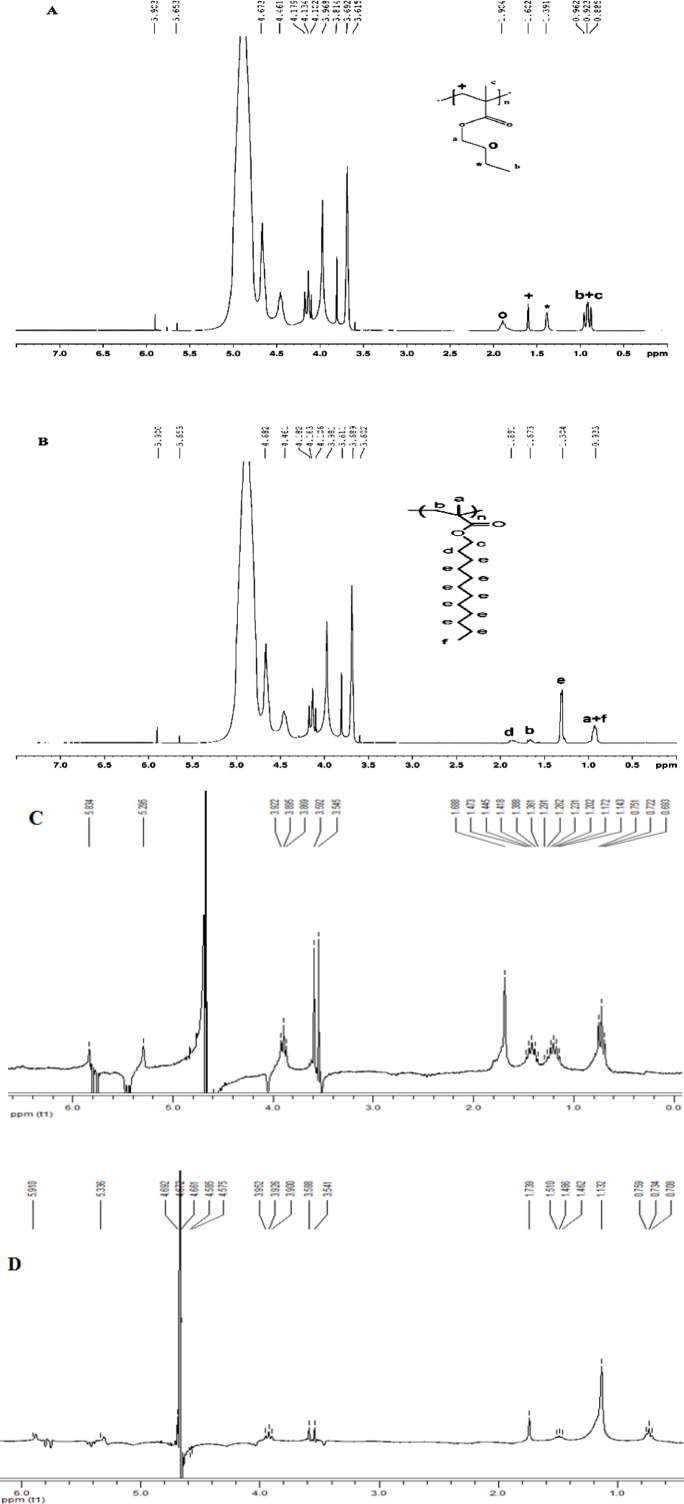
^1^H-NMR spectrum of ALG-B24 copolymer (A) and ALG-L12 copolymer (B), ALG+BMC physical mixture (C), ALG+LMC physical mixture (D)

LMC: lauryl methacrylate; ALG: alginate; BMC: butyl methacrylate

The ^1^H-NMR spectra of ALG-B24, ALG-L12 copolymers, and ALG+BMC and ALG+LMC physical mixtures were presented in Figure 3. 

^1^ **H-NMR (400 MHZ, D**_2_ **O)**

δ 0.7-1.1 (6H, butyl methacrylate: CH_3_), δ 1.2-2 (6H, butyl methacrylate: CH_2_), δ 4.05-4.2 (2H, butyl methacrylate: O-CH_2_), δ 4.3-5.2 (4H, that δ 5.0: alginate anomeric proton of Guluronate, δ 4.7: alginate anomeric proton of Mannuronate & H-5 of Guluronate-co-Mannuronate, δ 4.45: alginate H-5 of Guluronate-co-Guluronate)

^1^ **H-NMR (400 MHZ, D**_2_ **O)**

δ 0.7-1.1 (6H, lauryl methacrylate: CH_3_), δ 1.2-2 (22H, lauryl methacrylate: CH_2_), δ 3.9-4.1 (2H, lauryl methacrylate: O-CH_2_), δ 4.3-5.2 (4H, that δ 5.0: alginate anomeric proton of Guluronate, δ 4.7: alginate anomeric proton of Mannuronate & H-5 of Guluronate-co-Mannuronate, δ 4.45: alginate H-5 of Guluronate-co-Guluronate)

***Thermoanalysis***

**Figure 4:**
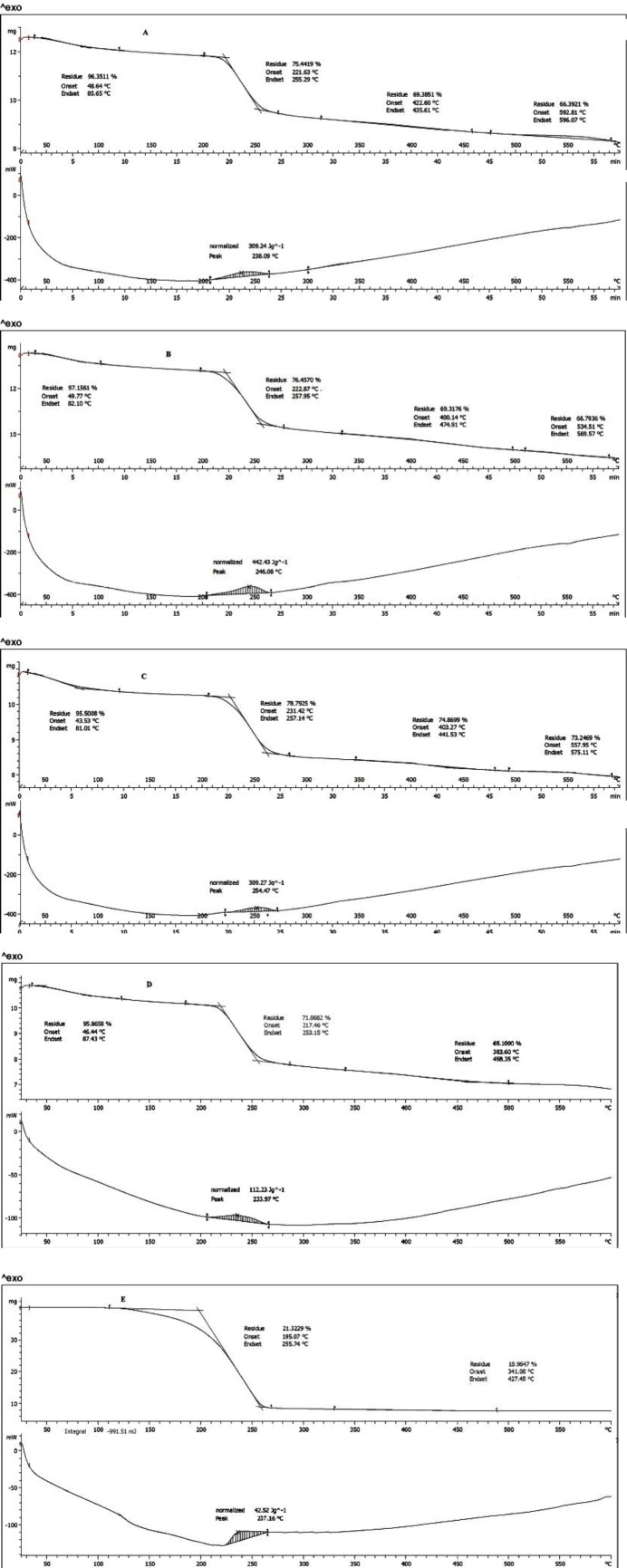
TGA thermogram of ALG (A), ALG-B24 (B), ALG-L12 (C), ALG+BMC physical mixture (D), and ALG+LMC physical mixture (E)

LMC: lauryl methacrylate; ALG: alginate; BMC: butyl methacrylate

As seen in Figure 4, thermoanalysis was performed for ALG, ALG-B24, and ALG-L12, ALG+BMC, and ALG+LMC physical mixtures. According to the DTA results, the peaks were assumed as the temperature of polymer chain breaking which were at temperatures 238, 246, 254, 234, and 237 °C for ALG, ALG-B24, ALG-L12, ALG+BMC, and ALG+LMC physical mixtures, respectively. 

The weight loss percentage of polymers according to TGA is reported in Table 1. The weight loss percentage of polymers according to TGA was 20.91, 20.70, 16.71%, 21.32, and 71.86% for ALG, ALG-B24, ALG-L12, ALG+BMC, and ALG+LMC physical mixtures, respectively. 

***Ft-IR***

**Figure 5:**
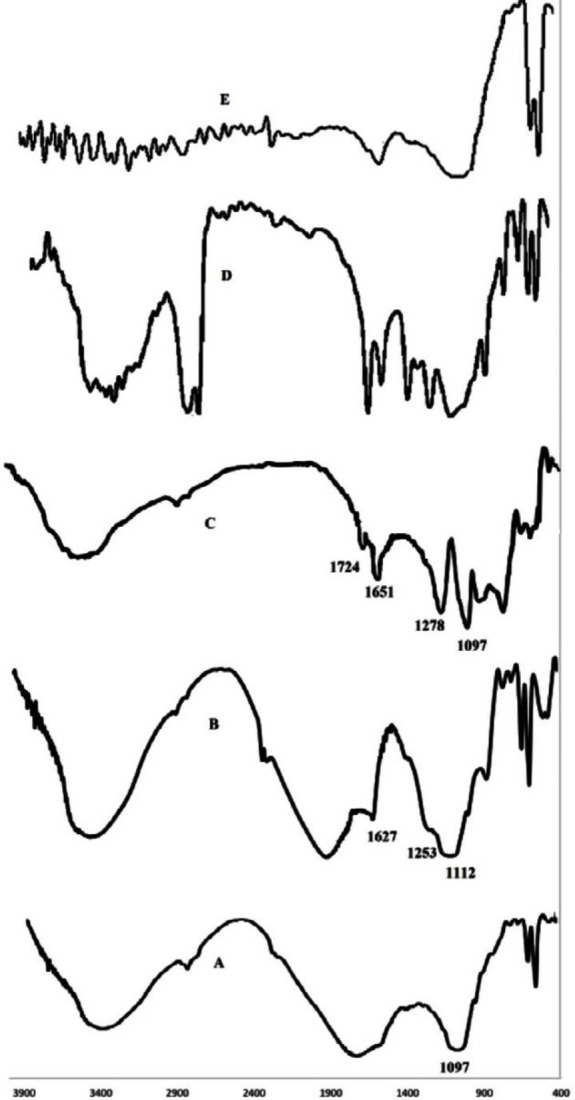
Ft-IR spectrum of ALG (A), ALG-B24 (B), ALG-L12 (C), ALG+LMC physical mixture (D), and ALG+BMC physical mixture (E)

LMC: lauryl methacrylate; ALG: alginate; BMC: butyl methacrylate

Figure 5 indicates the Ft-IR spectra of ALG, ALG-B24, and ALG-L12 with different characteristic peaks of polymers. 

***LNZ analysis***

LNZ quantification was performed at maximum absorbance wavelength at 251 nm in PBS, pH 7.4. 

***Analysis method validation***

According to the plotted standard curves, the resulting line has a regression coefficient (0.9993) close to 1, suggesting the linearity of the data. The equation related to the line was calculated as Y = 0.0479x + 0.3451*. *Inter, intra-day precision, and accuracy were 99.3±0.5, 99.6±0.3%, and 98.1±1.8%, respectively. The method sensitivity was evaluated by LOD and LOQ, which were 0.27 µg/ml and 0.81 µg/ml, respectively.

***Ocular insert preparation***

**Figure 6:**
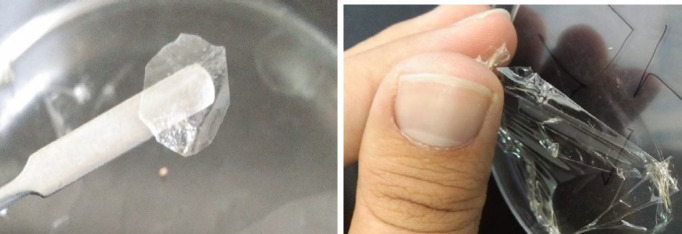
ALG-L12-LNZ insert, film formation (left), film separation (right)

ALG: alginate; LNZ: Linezolid

Considering cell cytotoxicity results, two ALG-B24 and ALG-L12 copolymers were selected to prepare ocular inserts using the solvent casting method. For better comparison, ALG inserts were prepared with the same method. Figure 6 shows the ALG-L12-LNZ insert. 

***Ocular inserts evaluation***

***Thickness measurement***

The thickness of ALG-B24-LNZ and ALG-L12-LNZ inserts was recorded as millimeters. Results are reported in Table 2.

***pH determination***

The aqueous solution of dissolved ALG-B24-LNZ and ALG-L12-LNZ inserts was used to determine inserts’ pH. All experiments were repeated in triplicate. Results are reported in Table 2. 

***Drug content***

The LNZ content of ALG-B24-LNZ and ALG-L12-LNZ inserts was determined. All experiments were tested in triplicate. Results are reported as drug content% in Table 2. 

***LNZ in vitro release***

**Figure 7:**
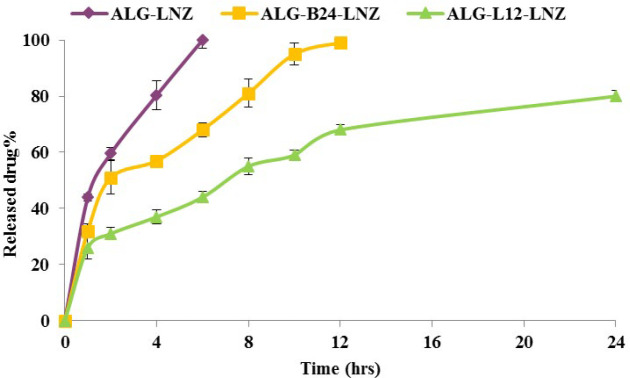
*In vitro *release profile of ALG-LNZ, ALG-B24-LNZ, and ALG-L12-LNZ

ALG: alginate; LNZ: Linezolid

The LNZ release process was investigated in PBS, pH 7.4. As seen in Figure 7, predictably, ALG-LNZ insert released the whole LNZ within 6 hr. LNZ release from ALG-B24-LNZ lasted 12 hr while ALG-L12-LNZ insert released 70 and 80% of LNZ in a slower manner within 12 and 24 hr. Release kinetic results were reported in Table 3.

***Mechanical properties***

The tensile strength, adhesiveness, and hardness were measured as mechanical properties of inserts. Results were reported in Table 2. The tensile strength of ALG-B24-LNZ and ALG-L12-LNZ was significantly higher (≈ 10 times) than that of the ALG-LNZ (*P*<0.05). The adhesive force of ALG-L12-LNZ was similar to ALG-LNZ and was higher (≈ 2 times) than ALG-B24-LNZ. Comparing ALG-LNZ, ALG-B24-LNZ hardness was significantly (*P*<0.05) reduced (≈11 times) while ALG-L12-LNZ showed higher hardness (≈1.5 times).

***Antibacterial efficacy***

MRSA detection results indicated the inhibition zone of 1.1±0.1 cm for the clinically isolated *S. aureus,* which confirmed that it was MRSA. The antibacterial efficacy of ALG-L12-LNZ insert as optimized formulation on MRSA microorganism as a resistant bacterium was assessed. No inhibition zone was observed in the blank samples after 24 hr. The inhibition zone of ALG-L12-LNZ and LNZ solution (positive control) after 4, 12, and 24 hr were reported in Table 4. 

## Discussion

Ocular inserts are attractive drug delivery systems since they can eliminate conventional ocular delivery obstacles such as rapid precorneal clearance and short residence time (30, 31). Sodium alginate is a natural and non-toxic polymer with mucoadhesive properties. Accordingly, due to its availability, non-allergic nature, biocompatibility, and low systematic absorption; sodium alginate can be used in ocular formulations. Alginate with a three-dimensional structure forms a stable gel in the presence of positive ions including calcium (8); however, it shows a fast release of its loaded drug. To overcome this problem, grafting appears as a suitable solution. One of the advantages of alginate grafts compared with intact polymers is their higher ability to control the release of their loaded drug (19). 

Considering these advantages, alginate grafted copolymers were synthesized in the present study (Figure 1) and their safety characteristics were evaluated. Cell cytotoxicity results (Figure 2) indicated significantly (*P*≤0.05) lower toxic effects for ALG-L12 and ALG-B24 compared with ALG-L24 and ALG-B12. Unlike the longer synthesis time and greater chance for side reaction series occurrence due to the grafted molecule breakdown and free radical formation (32), the synthesized alginate copolymers showed slight cytotoxicity against the HEK-293 cell line. Previous reports indicated higher toxicity against the HeLa S3 cell line for LMC compared with BMC, considering its higher log P and hydrophobic characteristics; however, it depends on the cells’ species (33). 

^1^H-NMR showed the successful grafting of sodium alginate with BMC and LMC. As shown in Figure 3, peaks in 0.5-2 ppm were related to BMC and LMC, while alginate peaks were detected in 3.5-5 ppm. All characteristic bands regarding ALG-B24 and ALG-L12 clearly appeared while as seen in Figure 3, the shape and intensity of ALG+BMC and ALG+LMC physical mixture peaks were not obvious which might be due to different solubility of ALG, BMC, and LMC in D_2_O. The in-proper baseline with negative peaks may confirm this theory also.

A number of weight change phases can be seen in the TGA thermogram. The first phase was related to the polymer moisture loss occurring at temperatures up to 100 °C. In the second phase, the polymer chains were fragmented and followed by the destruction of the polymer skeleton in the third phase (34). It was shown the major change in polymers’ weight occurs at the temperature of about 250 °C. The peak in the DTA thermogram for ALG was approximately at 238 °C and the corresponding values for ALG-B24 and ALG-L12 copolymers were 246 and 255 °C, respectively, which showed 12 and 17-degree increase in the ALG-B24 and ALG-L12 destruction temperature outsets (Figure 4). This suggests the higher interaction power of the polymer in the two grafted structures compared with the alginate (35). However, DTA thermogram peaks in ALG+BMC and ALG+LMC physical mixture were approximately at 234 and 237 °C, which were lower than ALG. On the other hand, the weight loss percentage in the initial dehydration phase for ALG-L12 was the lowest (16.7%) which may indicate higher thermal stability (20). Also, the required energy for the destruction of ALG, ALG-B24, and ALG-L12 was 309, 442, and 309 mj while ALG+BMC and ALG+LMC physical mixture showed most destruction with 112 and 42.5 mj energy. This higher required destruction energy for ALG-B24 and ALG-L12 versus physical mixture confirms that the conjugation process was performed.

ALG characteristic peaks considering C-O-C stretching vibrations of the saccharide structure (1097) in Figure 5A was presented in ALG-B24 and ALG-L12 grafted copolymers also; however, new steric carbonyl bond of ALG-B24 and ALG-L12 at 1253 and 1278 cm^-1^ and stretch carbonyl bond at 1627 and 1651 cm^-1 ^(Figures 5B and 5C) may confirm the successful grafting of ALG with BMC and LMC. ALG+BMC and ALG+LMC physical mixture Ft-IR peaks in Figures 5D and 5E, completely showed ALG, BMC, and LMC characteristic peaks.

LNZ analysis results suggested that the two used ALG-B24 and ALG-L12 copolymers had no intervening peaks at the wavelength of 251 nm as the LNZ absorption wavelength. In addition, the validation results of the LNZ assay confirmed that the analysis method was sensitive and completely validated with acceptable linearity, precision, and accuracy. 

In preliminary insert formulation design, ALG-B24 and ALG-L12 copolymers showed no good plasticity properties and were not easily separated from the plate. Therefore, glycerin was used as the plasticizer in the insert formulation to remove the problem (Figure 6). Also, ALG-B24 and ALG-L12 copolymers 2% w/v in combination with 10% w/v glycerin were optimized as the final formulation. Using calcium chloride as the most widely used agent that cross-links alginates (36), the insert formulation was easily removed from the plate. The results indicated acceptable insert thickness, which would not result in irritation in the cul-de-sac. As shown in Table 2, the pH of the formulations was within the physiological pH of the eye, which is not harmful to the cornea. LNZ content in all insert formulations varies from 98±3 to 110±5%, which showed an acceptable consistency indicating the repeatability of the production process (37). 

It was also shown (Figure 7) that LNZ release from ALG-B24-LNZ and ALG-L12-LNZ formulations was slower than from ALG-LNZ. ALG released the whole LNZ within 6 hr, while ALG-B24-LNZ released it after 12 hr. ALG-L12-LNZ showed a slower manner which released 70% and 80% of LNZ, within 12 and 24 hr, respectively. This points to the soundness of the synthesis reaction as BMC and LMC, when linked to ALG, created a lipophilic substrate that delayed the release process (38); however, due to release kinetics results in Table 3, it seems that ALG-LNZ and ALG-B24-LNZ release kinetic was best fitted with Higuchi while ALG-L12-LNZ showed zero-order kinetics. Since the ideal release pattern for slow and delayed drug delivery especially antibiotics is zero-order (39), it seems the final optimized formulation ALG-L12-LNZ with zero-order release kinetics may be a suitable carrier for LNZ.

The tensile strength is the resistive power of an insert against rupture. The tensile strength of ALG was lower than those of other grafted formulations, as was the case in the previous studies (40, 41). The tensile strength of ALG-B24-LNZ and ALG-L12-LNZ formulations was almost 10 times greater than that of the ALG-LNZ, showing the better strength properties in the selected formulations. 

Mucoadhesion is one of the most important and specific properties of sodium alginate, especially for new ocular drug delivery systems. In the present study, adhesion test was evaluated and the results indicated acceptable adhesive forces for ALG inserts produced in this study. However, it seems that unlike LMC, ALG grafting by BMC may reduce the adhesion properties up to 2 times. The ALG-L12-LNZ formulation showed a comparable adhesive force with ALG-LNZ, which was stronger (2 times) than ALG-B24-LNZ. 

Hardness is one of the most important properties of a thin film, which indicates its resistance to penetration and may present the useful life of the thin film, and especially its delamination (42). As shown in Table 2, the ALG-L12-LNZ formulation showed the highest hardness compared with ALG and ALG-B24-LNZ. 

Considering mechanical results, it seems that ALG-L12-LNZ with suitable tensile strength, adhesiveness, and hardness was able to more efficiently tolerate the applied pressure compared with the ALG-LNZ pointing to a good linkage between ALG and LMC, which formed a polymer network with good properties. 

In antibacterial experiments, MRSA detection was done using a disk diffusion test at a LNZ concentration of 30 µg/ml. The inhibition zone diameters of < 21 mm were considered as MRSA and those with zone diameters of >22 mm were considered to be sensitive (29). Considering the inhibition zone of 1.1±0.1 cm, MRSA detection was confirmed. The optimized ALG-L12-LNZ insert antibacterial efficacy on MRSA after 4, 12, and 24 hr (Table 4) was in an increasing manner, which may be attributed to the slower LNZ release from ALG-L12-LNZ. By the way, ALG-L12-LNZ insert showed comparable efficacy against MRSA microorganism with LNZ solution after 24 hr while within this time it released 80% of loaded LNZ. 

ALG: alginate

**Table 1:** Weight loss percentage of polymers according to the TGA chart

	Stage	Temperature difference (° C)	Weight Loss (%)	Phenomenon
ALG	Initial dehydration	85-200	3.65	Loss of moisture
	Most destruction	221-257	20.91	Chain fragmentation
ALG-B24	Initial dehydration	85-200	2.85	Loss of moisture
	Most destruction	223-258	20.70	Chain fragmentation
ALG-L12	Initial dehydration	85-200	4.5	Loss of moisture
	Most destruction	221-257	16.71	Chain fragmentation
ALG+BMC Physical mixture	Initial dehydration	46-87	3.5	Loss of moisture
	Most destruction	217-253	20	Chain fragmentation
ALG+LMC Physical mixture	Initial dehydration	-	-	-
	Most destruction	195-255	75.6	Chain fragmentation

LMC: lauryl methacrylate; ALG: alginate; BMC: butyl methacrylate

**Table 2:** Ocular inserts evaluation results

Formulation	**Thickness (mm)**	**pH**	**Drug content** **(%)**	**Tensile strength (MPa)**	**Adhesion force (g)**	**Hardness (g)**
ALG-LNZ	0.14±0.08	7.24±0.3	103±3	0.5±0.05	6.3±1.15	76±11.2
ALG-B24-LNZ	0.11±0.04	7.32±0.1	106±5	4.59±0.62	3.3±0.58	7±1.53
ALG-L12-LNZ	0.12±0.05	7.43±0.2	98±3	4.77±0.14	5.5±0.58	110±9.61

ALG: alginate; LNZ: Linezolid

**Table 3:** Ocular inserts release kinetic results

	Zero	First	Higuchi
ALG-	0.9925	0.9599	0.9981
ALG-B24-LNZ	0.9383	0.8954	0.9755
ALG-L12-LNZ	0.9934	0.8052	0.9697

ALG: alginate; LNZ: Linezolid

**Table 4:** Ocular insert antimicrobial results

	Inhibition zone (cm)		
Time (hr)	ALG-LNZ	ALG-L12-LNZ	LNZ solution
4	4.6±0.2	2.3±0.2	4.9±0.1
12	4.7±0.1	3.3±0.1	5.0±0.2
24	4.8±0.2	4.8±0.1	5.0±0.1

ALG: alginate; LNZ: Linezolid

## Conclusion

The present study aimed to develop a new lipophilic alginate-based ocular insert for the sustained release of LNZ. ALG-g-BMC and ALG-g-LMC co-polymer syntheses were confirmed using NMR, Ft-IR, and TGA techniques. A simple solvent casting method was used to produce ocular inserts. This method is economically and industrially affordable and can be scaled up. Given the cytotoxicity results, ALG-L12 and ALG-B24 co-polymers, which were safer, underwent the production and insert assessment phases compared with ALG. The optimized ALG-L12-LNZ inserts showed slower LNZ release than ALG-LNZ with a zero-order release kinetic, which may be an ideal release pattern for sustained drug delivery, especially for antibiotics. In addition, ALG-L12-LNZ’s better mechanical properties with suitable antibacterial efficacy on MRSA bacterium intensifies its capability as a good alternative carrier with less frequent administration for ocular solutions. 
